# Socioeconomic Factors in Adherence to HIV Therapy in Low- and Middle-income Countries

**DOI:** 10.3329/jhpn.v31i2.16379

**Published:** 2013-06

**Authors:** Karl Peltzer, Supa Pengpid

**Affiliations:** ^1^HIV/AIDS/SIT/and TB (HAST), Human Sciences Research Council, Pretoria, South Africa;; ^2^Department of Psychology, University of Limpopo, Turfloop, South Africa;; ^3^ASEAN Institute for Health Development, Madidol University, Salaya, Phutthamonthon, Nakhonpathom, Thailand 73170

**Keywords:** Antiretroviral therapy, highly active, Education, Employment, Income, Occupations, Social class

## Abstract

It is not clear what effect socioeconomic factors have on adherence to antiretroviral therapy (ART) among patients in low- and middle-income countries.  We performed a systematic review of the association of socioeconomic status (SES) with adherence to treatment of patients with HIV/AIDS in low- and middle-income countries. We searched electronic databases to identify studies concerning SES and HIV/AIDS and collected data on the association between various determinants of SES (income, education, occupation) and adherence to ART in low- and middle-income countries. From 252 potentially-relevant articles initially identified, 62 original studies were reviewed in detail, which contained data evaluating the association between SES and adherence to treatment of patients with HIV/AIDS. Income, level of education, and employment/occupational status were significantly and positively associated with the level of adherence in 15 studies (41.7%), 10 studies (20.4%), and 3 studies (11.1%) respectively out of 36, 49, and 27 studies reviewed. One study for income, four studies for education, and two studies for employment found a negative and significant association with adherence to ART. However, the aforementioned SES determinants were not found to be significantly associated with adherence in relation to 20 income-related (55.6%), 35 education-related (71.4%), 23 employment/occupational status-related (81.5%), and 2 SES-related (100%) studies. The systematic review of the available evidence does not provide conclusive support for the existence of a clear association between SES and adherence to ART among adult patients infected with HIV/AIDS in low- and middle-income countries. There seems to be a positive trend among components of SES (income, education, employment status) and adherence to antiretroviral therapy in many of the reviewed studies.

## INTRODUCTION

The clinical efficacy of antiretroviral therapy (ART) in suppressing the HIV virus and improving survival rates for those living with HIV has been well-documented (1-3). However, successful antiretroviral therapy is dependent on sustaining high levels of adherence (correct dosage, taken on time, and in the correct way—either with or without food). The minimum level of adherence required for antiretroviral drugs to work effectively is 95% (4). Although more potent antiretroviral regimens can allow for effective viral suppression at moderate levels of adherence, no or partial adherence can lead to the development of drug-resistant strains of the virus (5-7). Adherence to ART is influenced by factors associated with the patient, the disease, the therapy, and the relationship of the patient with healthcare provider (8-10). Patient-related factors include socioeconomic status (SES) (8,10).

A review of studies since 2005 on SES and adherence to ART primarily in high-income countries, did not provide conclusive support for a clear association between SES and adherence (8). However, it is not clear what effect socioeconomic factors have on adherence to ART in low- and middle-income countries. A possible association between SES and adherence to ART among HIV patients may have an impact on the success of their treatment (8,10).

## MATERIALS AND METHODS

### Literature search

We performed a systematic search of the literature to identify reviews and original studies that reported data on the impact of SES on adherence to ART. The relevant studies were identified by the use of electronic databases, such as MEDLINE, EMBASE, SCI Web or Science, NLM Gateway, and Google Scholar. The last search was conducted in November 2011. In addition, relevant articles from the list of references of the initially-retrieved papers were identified. Studies conducted only in low- and middle-income countries were included, according to World Bank classifications (11). Five different search strategies using the following key words were employed: (i) Socioeconomic status AND (HIV OR AIDS) AND (compliance OR adherence), (ii) (Compliance OR adherence) AND (HIV OR AIDS) AND determinants, (iii) (AIDS OR HIV) AND (compliance OR adherence) AND education AND/OR income AND/OR occupation, (iv) (AIDS OR HIV) AND (compliance OR adherence) AND determinants, and (v) (AIDS OR HIV) AND (compliance OR adherence).

Defining socioeconomic status (SES) is difficult because a single, consistent unit of measurement was not used in the studies reviewed. Further, a debate exists in the public-health arena on the appropriate components of socioeconomic status and methods of measurement (12). Krieger *et al*. (13) have argued that it is important to distinguish two different components of socioeconomic position (actual resources and prestige or rank-related characteristics), and they preferred the use of the term ‘socioeconomic position’ instead of ‘socioeconomic status’. In addition, they argued that it is important to collect data at the individual, household and neighbourhood level (12,13). Additional points emphasized included that data on individuals supported from ‘annual family income’ should be collected, measurements should incorporate the recognition that socioeconomic position can change over a lifetime, and measures of socioeconomic position may perform differentially based on racial/ethnic group and gender background (12,13). Most of the reviewed articles did not attend to these complexities, rather used one to three measures of SES, most often simplistic measures of income, education, and occupation or employment status. The reviewed articles were analyzed with the understanding that the complexities present in SES highlighted by Krieger *et al*. (13) should ideally be incorporated in future studies designed to tease out the relationship between SES and adherence to ART in low- and middle-income populations. Meanwhile, the term SES is used in this article rather than socioeconomic position, simply because this is how these measures were discussed by the authors in the papers reviewed (12). SES reflects different aspects of social stratification, and the traditional indicators at the individual level have been income, education, and occupation (14,15). There is no single-best indicator of SES suitable for all study objectives and applicable at all time-points in all settings. Each indicator measures different, often related aspects of socioeconomic stratification and may be more or less relevant to different health outcomes and at different stages in the course of life (15). Galobardes *et al*. (16) described the theoretical basis of the following three indicators used for measuring SES:

(a) Education attempts to capture the knowledge-related assets of a person. As formal education is normally completed in young adulthood and is strongly determined by parental characteristics, it can be conceptualized within a course of life framework as an indicator that, in part, measures socioeconomic position (SEP) in early life (16).
(b) Income is the indicator of SEP that most directly measures the material resources component (16).
(c) Occupation represents Weber's notion of SEP as a reflection of a person's place in society relating to their social standing, income, and intellect (16).

### Selection of studies

The inclusion and exclusion criteria used for the reviewed studies were set before the literature search. Studies included in our study concerned only individual HIV-infected adult patients and their adherence to antiretroviral therapy. Reviews and editorials were not included in our systematic review. Studies that focused on HIV-infected illicit and/or licit drug-users and/or those with severe mental illness were excluded since such persons may need more creative approaches than other patients to ART adherence that differentiates them from the general population (8,17-19). Two authors of the present article evaluated the eligibility studies obtained from the literature search using a predefined protocol. The two authors worked independently to scan all abstracts and obtained full-text articles. In cases of discrepancy, agreement was reached by consensus.

### Data extraction

Two authors of the present article independently extracted and compiled the data. For each identified study that met the selection criteria, details were extracted on study design, characteristics of study population, data relevant to SES, the measure of adherence, the overall adherence, and findings regarding the association between determinants of SES and adherence on to an Excel spreadsheet. In this review, three parameters as major factors contributing to SES were assessed, namely income, education, occupation/employment status and their association with adherence to ART.

The following diagram presents the various steps in the process of selecting studies.

**Figure.**
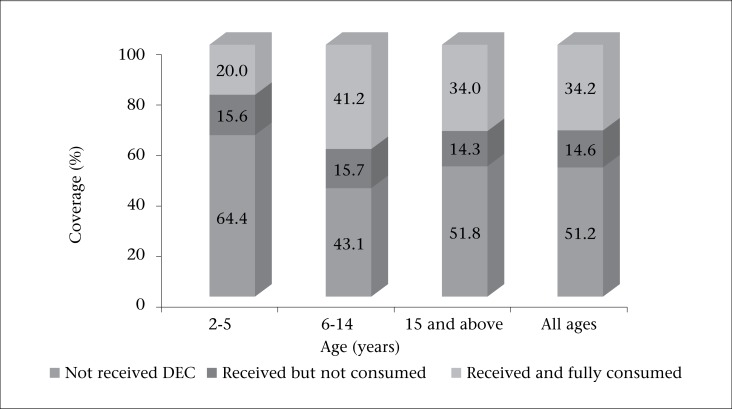
Flow-diagram of reviewed studies

## RESULTS AND DISCUSSION

The literature search identified 252 potentially-relevant studies, from which we further reviewed 62 studies with original data. In Annexure A-F, the characteristics of 62 studies that were included in the systematic review are presented by region and country. The year of publication of the studies ranged from 2002 to 2011. There was considerable variability across the studies in setting and patient population, largely because these were conducted in different low-resource settings, with different cultures, incomes, and education levels (Table 1).

**Table 1.** Education and income (country indicators) in study countries

Country	Education		Income	
	Adult literacy (%)	Primary school enrollment rate: Male/Female	Gross national income per capita (PPP int. $)	Living on <1$ (PPP int. $) a day (%)
Botswana	83	86/88	12,840	-
Brazil	90	95/93	10,200	5.2
Burkina Faso	29	67/59	1,170	56.5
Cameroon	76	97/86	2,190	32.1
China	94	-	6,890	15.9
Columbia	93	93/80	8,600	16.0
Costa Rica	96	-	10,930	2.0
Cuba	100	99/99	-	-
Dominican Republic	88	92/82	8,110	4.4
Ethiopia	36	85/80	930	39.0
India	63	91/88	3,250	41.6
Ivory Coast	55	62/52	1,640	23.3
Jamaica	86	82/79	7,230	<2.0
Kenya	87	82/83	1,570	19.7
Mali	26	79/66	1,190	51.4
Nigeria	60	64/58	2,070	64.4
Papua New Guinea	60	-	2,260	-
Rwanda	70	95/97	1,060	76.6
Senegal	42	72/74	1,810	33.5
South Africa	95	87/88	10,050	26.2
The Gambia	45	67/71	1,330	34.3
Thailand	94	91/89	7,640	<2.0
Uganda	75	96/99	1,190	51.5
United Republic of Tanzania	73	96/97	1,350	88.5
Zambia	71	96/92	1,280	64.3

Source: World health statistics 2011 20

**Table 2.** Summary of studies on the association between the main components of socioeconomic status and adherence to antiretroviral therapy

SES component	Number of studies N	Positive association N (%)	Negative association N (%)	No association N (%)
Education	49	10 (20.4)	4 (8.2)	35 (71.4)
Income	36	15 (41.7)	1 (2.8)	20 (55.6)
Occupation/employment	27	3 (11.1)	2 (7.4)	22 (81.5)
SES	2	0	0	2 (100)

Regarding the study design, 44 cross-sectional (21,24,26,28-31,33-37,41,42,47-49,53,55,56,58-72,74-76,78-82), 19 longitudinal (22,25,27,32,38-40,43-46,50-52,54,57,77), and two case-control (23,73) studies were included in the review. The average number of patients was 400 per study in the total of 62 studies (ranging from 53 to 2,381, depending on the study setting).

Studies varied in the measurement of adherence (pills per dose, doses per day, days of treatment per week, time schedule for pill-refill, etc.) and used different cutoff points of adherence (from 80% to 100% of dosage) to dichotomize the patients between adherence and non-adherence to ART. Two studies focused directly on the association between SES or its main determinants analyzed as a group and adherence (40,78). The available reported data regarding the method, with which adherence to antiretroviral treatment was measured, and the data on overall adherence are presented in Annexure A-F. In 50 out of 62 studies included in the review, self-report by the patients was the main measure of adherence to treatment (21,22,24,26,27,29-32,34-37,39,41,42,44-49,51,53,56,58-69,70-82); six studies used pill counts, MEMS, pharmacy refills as the main measures (23,40,43,54,55,57), and in six studies both self-report and objective adherence measures (25,28,33,38,50,52) were used.

The main parameters affecting SES (income, education, occupation) were only examined as a group comprising SES in two studies but, in 61 studies, these were rather regarded as socioeconomic characteristics. Therefore, many studies lacked data concerning some of the parameters. There were insufficient data regarding income in 26 studies (22,28,29,31,33,37,38,41,47,48,50,51,53,54,56,60,68-70,72,74,75,80-82) and educational level in 14 (26,28,30,37,39-41,46,59,61,62,65,68) of the 62 reviewed studies (Some of the studies had data on income but not on education, and others had the reverse). Employment and/or occupational status was assessed in 28 studies (22-24,28,29,31,34-37,39-42,44,45,53,54,58,59,61,67-69,70,77,78,81,82). However, no data were given on occupational status or working position in 18 of those 28 studies.

The main findings regarding the analysis of the association of SES or the various components of SES and adherence were as follows: income, level of education, and employment/occupational status were significantly and positively associated with the level of adherence in 15 studies (41.7%) (21,24,26,32,39,43,46,49,62,63,65-67,76,78), 10 studies (20.4%) (33,35,53,66,69,71-73,75,77), and three studies (11.1%) (28,29,77) respectively out of 36, 49, and 27 studies reviewed. Most significant findings refer to a positive association between levels of SES components and levels of adherence to antiretroviral treatment, although one for income (59), four for education (21,31,43,63) and two for employment (59,77) of the reviewed studies suggest an inverse association with adherence. However, the aforementioned SES determinants were not found to be significantly associated with adherence in relation to 20 income-related studies (71,73,23,24,25,30,34-36,42,43,45,57,61,77), 35 education-related studies  (22-25,27,29,32,34,36,38,42,44,45,47-52,60,64,67,70,74,76,78-81,82),   22   employment/occupational status-related studies (22-24,34-36,41,42,44,45,49,53,54,58,67-70,78,79,81,82) and two SES-related studies (40,78) (Table 2).

### Limitations

This systematic review has several limitations. First, it was not possible to make a synthesis of the data, using the principles of meta-analysis due to the fact that there was considerable heterogeneity among the reviewed studies. Adherence was measured by different methods in each of the studies and the cutoff percentage of adherence to treatment between ‘adherent’ and ‘non-adherent’ varied among the studies. Another limitation was that the majority of the studies examined the used unreliable measures of adherence (self-report, in particular) as the adherence outcome measure. In addition, SES was not focused upon as a homogenous group of specific factors in most of the reviewed studies but was rather dispersed among its components, which were regarded as socioeconomic information. Therefore, partial data had to be collected regarding the association of such SES components, and adherence to antiretroviral therapy, where and if such an association was assessed. Occupation was mainly assessed in terms of employment status because often no data were given on status of occupation or working position of the patients (8).

### Conclusions

The systematic review of the available evidence found a positive trend among components of SES (income, education, occupation/employment) and adherence to antiretroviral therapy in many of the reviewed studies. However, we found inconclusive support for a clear association between SES and adherence among patients infected with HIV/AIDS in low- and middle-income countries. The association between SES and adherence may differ depending on the cultural/economic/geographic context of the countries studied, and results emphasize a site-specific approach to adherence studies and programmes. Future studies should measure socioeconomic factors more accurately and, thus, may further explain the different impacts of SES to ART adherence. In the absence of a gold standard for measure of adherence, future studies should assess many outcomes.

**Annexure A.** Impact of socioeconomic factors on HAART adherence among adults: study characteristics (Africa 1)

Country of study, year of publication, first author (Reference number)	Study design, setting	Study population, sample-size, type of medication	Adherence: measurement, definition, and total adherence	Socioeconomic status	Impact of SES on adherence
Botswana 1 [2003] Weiser (21)	Cross- sectional study	109 patients, 40 patients combination therapy with 2 nucleoside reverse transcriptase inhibitors (NRTIs) and 1 protease inhibitor, 2 NRTIs and 1 non-NRTI, or 2 protease inhibitors	Self-reported adherence over the previous day, week, month, and year; 54% of patients were adherent (≥95% by self-report while 56% were adherent by providers’ assessment	Secondary school or more: 87%; From those who missed treatment, 48% said because of finances	Cost is a barrier to treatment: AOR=0.15, 0.06–0.35; Incomplete secondary education: AOR=3.87, 1.21–12.40
Botswana 2 [2010] Do (22)	Cross-sectional prospective survey; Outpatient adult infectious disease clinic, Gaborone	300 adult patients CBV/NVP: 66.0%; CBV/EFV: 25.7%	Self-reported, institutional adherence, and a culturally-modified Morisky scale; The overall ART adherence rate was 81.3% based on 4-day and 1-month patient recall and on clinic attendance for ARV medication refills during the previous 3 months	Unemployed: 44.3%; Secondary education: 55.7%	Level of education: NS; Employment status: NS
Botswana 3 [2011] Gust (23)	Case-control study; 8 public health urban clinics	252 adherent patients; 127 non-adherent patients	Pharmacy refill visits; Criterion of attending ≥80% of visits within 6-month period	Secondary education: 54.6%; Employed: 63.7%; Income per month (Pula: 0-900): 48.8%	Education: NS; Employment status: NS; Income: NS
Cameroon 1 [2009] Boyer (24)	Cross- sectional study; 6 public hospitals	532 patients	Self-reported dosie-taking during the prior 4 days and dosing time schedule in the past 4 weeks; the 53.9% to 100% adherent in dose-taking in the past 4 days and dosing time schedule in the past 4 weeks	20% financial difficulty in purchasing their ARVs; Completed primary education: 55.6%; Monthly household income (median): US$ 128; Having economic activity: 70.8%	Difficulty in buying ARV: OR=0.24 (0.15-0.4); Education: NS; Household income: NS; Having economic activity: NS
Cameroon 2 [2009] Rougemont (25)	Longitudinal study; Central Hospital, Yaoundé	312 patients at the start of ART; Triple-drugs regimens consisting of two NRTIs and one non-NRTI	Self-reported adherence in the past month; 78% claimed not to have missed a single dose; Pharmacy-records review; 64% pharmacy-appointed dates adherence (renewal of prescription within 2 weeks after the scheduled date)	Monthly income of less than US$ 50: 46%; Secondary or more education: 65%	Monthly income: NS; Education: NS
Cameroon 3 [2011] Boyer (26)	Cross- sectional study; 6 hospitals	2,381 patients	Self-reported doses taken and compliance with the dosing schedule in the past 4 days; 56.6% good adherence	Financial difficulty in purchasing ARVs in previous 3 months	Non-adherence: Patients with financial difficulties
Cameroon 4 [2011] Roux (27)	Prospective cohort study	401 patients	Self-reported adherence in the past 4 days; 66% adherent (100% of doses in the past 4 days)	≥secondary education: 51%; Subjective social level scale: Median=2	Education: NS; Social level scale: NS
Ethiopia 1 [2009] Beyene (28)	Cross- sectional study	422 patients	Self-reported adherence assessment of 15 days; 93.1% (≥95%); Unannounced pill count method (n=90): 88.1% adherent (≥95%)	Unemployed: 59%	Unemployment: AOR=0.01, 0.00-0.29
Ethiopia 2 [2010] Giday (29)	Cross- sectional study	510 AIDS patients seen over one month	Self-report: 88.2% of them had ≥95% and 97.1% of them had ≥80% antiretroviral adherence rate over one month period	Occupation: 39.6% no job; No education: 13.5%	Having a job; Education: NS
Ethiopia 3 [2010] Tiyou (30)	Cross- sectional study	319 adults; HAART regimen of Stavudine (d4T), Lamivudine (3TC) and Nivirapine (NVP):71.8%	Self-reported adherence (not missing a single dose) based on the combined indicator of the dose, time and food in the past week was 72.4%	Median monthly income of the participants and their family: 300.00 and 350.00 Ethiopian Birr	Average family income: NS

95% Confidence intervals; AOR=Adjusted odds ratio; NS=Not significant; OR=Odds ratio; RH=Risk ratio

**Annexure B.** Impact of socioeconomic factors on HAART adherence among adults: study characteristics (Africa 2)

Country of study, year of publication, first author (Reference number)	Study design, setting	Study population, sample-size, type of medication	Adherence: measurement, definition, and total adherence	Socioeconomic status	Impact of SES on adherence
Ivory Coast [2007] Eholié (31)	Cross- sectional study; 3 urban HIV outpatient clinics	308 patients; Mean time on HAART: 22 months	Self-report of pill intake during the previous 7 days; The median self-reported adherence rate was 78%; 76% of patients considered incompletely adherent (adherence rate <90%)	Secondary school or higher: 73%	Non-adherence; School level; ≥secondary: AOR=1.88, 1.06 3.35
Kenya [2010] Unge (32)	Prospective open cohort study; African Medical Research Foundation (AMREF) Clinic in the Kibera slum	800 patients; First-line ART-regimens: Stavudine, Lamivudine, and Nevirapine/Efavirez; Second-line regimens, including Zidovudine, Abacavir, Didanosine, Ritonavir-boosted Lopinavir (Kaletra), and Tenofovir	Self-reported adherence in past 4 days; More than one-third of patients were non-adherent (<95%) when all three aspects of adherence—dosing, timing, and special instructions—were taken into account	Up to primary school: 60%; >5000 KSH income/month: 59.5%	Low adherence index: Living below poverty limit: AOR=3.28, 1.27-8.48; Low education: NS
Nigeria 1 [2005] Iliyasu (33)	Cross- sectional study; Aminu Kano Teaching Hospital, Kano	263 AIDS patients	Patient's reported consumption of antiretroviral drugs was compared with the physician's prescription in the 7-day period preceding the interview; Only 142 (54.0%) of the 263 respondents took at least 80% of the antiretroviral drugs prescribed. Sixty-one (23.2%) did not miss any dose of the drug	Tertiary education: 36.1%; Secondary education: 34.2%	Formal education: OR=3.97; (1.75–9.24) (univariate analysis only)
Country of study, year of publication, first author (Reference number)	Study design, setting	Study population, sample-size, type of medication	Adherence: measurement, definition, and total adherence	Socioeconomic status	Impact of SES on adherence
Nigeria 2 [2008] Shaahu (34)	Cross- sectional study	428 patients	Self-reported adherence rate was 268 (62.6%), measured as consistent use from onset of study period	Unskilled occupation: 70.6%; Post-secondary education: 41.1%; Monthly income ≥5,000 Naira: 40.9%	Occupation: NS; Education: NS; Monthly income: NS
Nigeria 3 [2009] Uzochukwu [35]	Cross- sectional study	174 patients on ART for at least 12 months	Self-reported missing of medication in the past month; 75% not adhering fully to their drug regimen	Occupation: Business/trading 39.6%, civil servant 18.4%, Unemployed: 11.5%; Head of household's income/month <5000: 48.3%; Years of formal education:Median=4.9 years	Non-adherence; Formal education; Coefficient=-0.26 (p=0.007); Employment status: NS; Household income: NS
Nigeria 4 [2010] Adewuya (36)	Cross- sectional study	182 persons with HIV infection	Self-reported Morisky Medication Adherence Questionnaire; 26.9% low adherence	Secondary-school education: 50.0%; Low SES (occupational status and income): 34.1%	Educational level: NS; SES: NS
Nigeria 5 [2010] Salami (37)	Cross- sectional study; Ilorin	253 adult patients	Self-reported past 30 days; 70.8% adherent (≥95%)	Employed: 95.7%	Employed: Spearman rho=0.59
Nigeria 6 [2010] Ukwe (38)	Prospective study	299 patients; HAART type: D4T + 3TC + NVP 219 (73.2%)	Self-reported adherence in past 7 days; 86.1% average adherence (≥95%) over 3-month assessment; Use of an adherence aid (pill box) was correlated with adherence	Secondary education: 45.5%	Education: NS

95% Confidence intervals; AOR=Adjusted odds ratio; NS=Not significant; OR=Odds ratio; RH=Risk ratio

**Annexure C.** Impact of socioeconomic factors on HAART adherence among adults: study characteristics (Africa 3)

Country of study, year of publication, first author (Reference number)	Study design, setting	Study population, sample-size, type of medication	Adherence: measurement, definition, and total adherence	Socioeconomic status	Impact of SES on adherence
Senegal [2003] Lanièce (39)	Prospective cohort study (2 years)	158 adults	Self-reported adherence in the past month; 69% optimal (100%) adherence; 91% mean overall adherence	Median monthly income:15,000 FCFA (about US$20) (50.6%); Not in paid employment: 41%	Free of charge ARVs during 17 months of the study
South Africa 1 [2003] Orrell (40)	Prospective cohort study; Public sector hospital, Cape Town	289 patients	Clinic-based pill counts and pharmacy refill data over 48 weeks; The median adherence of the cohort up to 48 weeks was 93.5%	Low socioeconomic status; (income, education, employment): 42%	Socioeconomic status: NS
South Africa 2 [2004] Nachega [41]	Cross- sectional study; Chris Hani Baragwanath Hospital, Soweto	66 patients; Median duration of ART use for 18 months	Self reported adherence; Adherence was >95% for 58 patients (88%) for previous month	Employed: 59.9%; SES (employment, tap-water, electricity, overcrowding; Score 0-4): Mean 3.2	Employment status: NS SES: NS
South Africa 3 [2008] Malangu (42)	Cross- sectional study	180 patients; Mean age of 36.7±8.1 years	Self-reported mean number of doses missed during the last seven days prior to the interview was 2.7±3.9; The mean adherence level was 92.3%	High school level of education: 73.9%; Unemployed: 86.7%; Received disability grants: 34.4%	Education: NS; Employment status: NS; Receiving a disability grant: NS
South Africa 4 [2010] Maqutu (43)	Prospective study	688 patients	Pharmacy-records (pill counts); During the first month of therapy, 79% of the patients were adherent (≥95%) to HAART	Secondary-school or higher level of education: 68%; Classified as a source of their household's income: 28%; Owned cell phones: 42%; No schooling: 12%	Cellphone ownership: AOR=1.26, 1.06-1.50; Urban treatment site: AOR=4.35, 2.26–8.37; No schooling: AOR=5.04, 1.84-13.82; Income: NS
South Africa 5 [2010] Peltzer (44)	Prospective cohort study (6 months); 3 hospitals, KwaZulu-Natal	735 patients	Two self-reported adherence measures; 30-day VAS at ≥95% adherent 82.9%; Self-reported 4-day recall dose adherence 84.5%	Grade 8 or higher formal education: 61.9%; Formal salary as main source of household income: 31.7%; Disability grant: 52.5%; Unemployed: 59.6%	Education: NS; Employment status: NS; Disability grant: NS; Urban residence: AOR=2.78, 1.60-4.83
South Africa 6 [2011] Peltzer (45)	Prospective cohort study (20 months)	735 patients; HIV medications for 76.3% patients included Lamivudine (3TC), Stavudine (d4T) + Efavirenz and for 23.7% Lamivudine (3TC), Stavudine (d4T) + Nevirapine	Self-reported adherence measure; At 12 and 20 months using the VAS: 89.6% and 91.6% adherent at ≥95%	Grade 8 or higher formal education: 61.9%; Formal salary as main source of household income: 31.7%; Disability grant: 52.5%; Unemployed: 59.6%	Income: NS; Education: NS; Employment status: NS; Urban residence: AOR=3.71, 1.56-8.83

95% Confidence intervals; AOR=Adjusted odds ratio; NS=Not significant; OR=Odds ratio; RH=Risk ratio

**Annexure D.** Impact of socioeconomic factors on HAART adherence among adults: study characteristics (Africa 4)

Country of study, year of publication, first author (Reference number)	Study design, setting	Study population, sample-size, type of medication	Adherence: measurement, definition, and total adherence	Socioeconomic status	Impact of SES on adherence
Tanzania 1 [2007] Ramadhani (46)	Cross- sectional cohort study	150 patients on ART for at least 6 months	Self-reported assessment on incomplete treatment adherence; 84% reported not missing any doses of ART from the start of treatment	Weekly ART expenditure per patient: Median USD (range) 18.1 (0–104.4); Duration of self-funded treatment, proportion of treatment duration: 0.12	Non-adherence: Paying for treatment AOR=23.5, 1.2-444.4); Weekly ART expenditure: NS
Tanzania 2 [2010] Watt (47)	Cross- sectional study	340 patients	Self-report; 94.1% reporting at least 95% adherence on both four-day and one-month self-report measures	Completed primary education only: 60.9%	Education: NS
The Gambia [2010] Hegazi (48)	Cross- sectional study	147 patients	Self-reported adherence; 31% reported missing 1-3 doses in the past month	No formal education: 38%	Illiteracy: NS
Uganda 1 [2005] Byakika-Tusiime (49)	Cross- sectional study	304 patients purchasing ART	Self-reported number of missed doses over the last three days; 44% reported having missed at least one dose of the ARVs in the previous three-month period	Post-secondary education: 63.2%; Monthly income: <500,000 USh (US$ 250): 87.8%	Non-adherence: Monthly, US$ 50: AOR=2.77, 1.64-4.67 Education: NS Employment: NS
Uganda 2 [2008] Abaasa (50)	Retrospective cohort study; TASO clinic, Kampala	897 patients	Self-report and pill count methods; 21.9% patients had a mean adherence of 95% or less	No education: 17.5%	Education: NS
Uganda 3 [2009] Bajunirwe (51)	Prospective cohort study; Kitagata Hospital	175 patients	3-day self-report to measure adherence; Patients were considered non-adherent if they missed at least 1 antiretroviral pill and 100% adherent if they had not; At baseline, 149 (85%) reported 100% adherence	Primary education: 53.1%	Non-adherence; Education: NS
Uganda 4 [2009] Byakika-Tusiime (52)	Longitudinal study	177 patients; 75 patients newly-initiating ART and 102 on stable ART	Unannounced pill counts; 3-day self-report and a 30-day visual analogue scale; Mean adherence was over 94%	Education >primary: 49.4%; Median monthly income: US$90	Education: NS; Income: NS
Uganda 5 [2009] Nakimuli-Mpungu (53)	Cross- sectional study	120 adult patients	Self-reported missed doses; 17.2% non-adherence (<90%) to HAART in the previous month	Secondary education: 32.8%; Employed: 65.6%	No education: OR=0.32, 0.12-0.85; Employment status: NS
Uganda 6 [2010] Kunutsor (54)	Prospective study over a 28-week period; district hospital	392 adult patients; Majority: first-line fixed-dose combination regimen: Zidovudine, Lamivudine, and Nevirapine or Stavudine, Lamivudine, and Nevirapine	Clinic-based pill count in the past 4 weeks; 98.8% mean medication adherence: 93.1% (≥95%) optimal medication adherence	Primary education or less: 73%; Unemployed: 55%	Education: NS; Employment status: NS
Zambia 1 [2008] Carlucci (55)	Cross-sectional survey, chart review; Macha Mission Hospital	424 patients	Pill counts; 83.7% had optimal (≥95%) adherence at the first month	>Primary education: 40%	Education: NS
Zambia 2 [2009] Birbeck (56)	Retrospective chart review	255 patients	Self-reported assessment; 59.2% good adherence (attended all scheduled ART clinic visits with no lapse in drug collection and no documentation indicating adherence problems)	Primary or less education: 54.9%	Education: NS
Zambia 3 [2011] Birbeck (57)	Prospective cohort study	496 adults	Pharmacy-records; Almost 60% had good adherence (no documented lapses in drug acquisition as per pharmacy-records, and no patient or healthcare worker reports of adherence problems)	Wealth in household goods; Median=US$ 1,078 (IQR=62-1,523) Food insecurity: 44.4%; Education (mean years): 7.2	Poor adherence; Wealth: NS; Food insecurity: NS; Education: NS
Burkina Faso and Mali [2007] Aboubacrine (58)	Cross- sectional study; Bamako (n=110) and Ouagadougou (n=160)	270 patients	Self-reported number of doses missed yesterday, the day before yesterday, and over the previous week; 58.5% of the patients reported having a complete ART adherence (‘always’ taking theirmedication)	High school education: 51.5%; Had no revenue or earned <US$ 54 per month: 54%; Occupation with salary: 49.2%	Education: NS; Occupation: NS; Income: NS
Kenya, Uganda, Zambia, Nigeria, and Rwanda [2010] Etienne (59)	Cross-sectional study	921 adult patients on ART for at least 1 year; NVP combination 59.3%; EFV combination 31.8%	Self-reported adherence; 72% adherent (not missed doses in the past week or missed appointments in the past 3 months)	Paid job: 44.5% Living in own home: 54.6%	Paid job: OR=0.67, 0.48-0.93; Own home: OR=1.48, 1.05-2.11

OR=Odds ratio; AOR=Adjusted odds ratio; 95% Confidence intervals; NS=Not significant; RH=Risk Ratio

**Annexure E.** Impact of socioeconomic factors on HAART adherence among adults: study characteristics (Asia 1)

Country of study, year of publication, first author (Reference number)	Study design, setting	Study population, sample-size, type of medication	Adherence: measurement, definition, and total adherence	Socioeconomic status	Impact of SES on adherence
China [2008]Wang (60)	Cross- sectional study; 7 free treatment sites	380 patients; 3-drug regimen	Adherence measured by CPCRA self-report: 79% taking 100%, (17%); 80-99%, and 4% (0-79%) in the past 7 days	Less than high school education: 84%	Urban/rural: NS; Level of education: NS
India 1 [2005]Safren (61)	Medical charts review, NGO, Chennai	304 patients with HIV	Self-report of missing doses; Skipping doses at least weekly=irregular (17.8%)	Most common reason for non-adherence: 32% (cost)	Monthly cost of regimen: NS
India 2 [2007]Wanchu (62)	Cross- sectional study; Chandigarh	200 patients (138 males) receiving generic triple drug reverse transcriptase inhibitor-based antiretroviral medications	Self-report; 147 did not miss any dose; Fifty-three (26.5%) missed at least one dose during the preceding 4 weeks	Bought the medications from their own resources: 35%	Non-adherence; Financial constraints
India 3 [2008]Sarna (63)	Cross- sectional study	310 patients; 80% first-line Nevirapine-based regimen [160 on Stavudine (D4T)/Lamivudine (3TC)/Nevirapine (NVP), and 112 on Zidovudine (ZDV)/3TC/NVP)]	Self-reported adherence based on a 4-day recall; Mean 4-day adherence was 93%	Clients without coverage were spending on average US$ 66 per month out-of-pocket for their treatment; Employed: 85%; Less than university education: 63%	Non-adherence; Free ARV vs paid out of-pocket: AOR=4.05, 1.42–11.54; 5 years education vs University: AOR=4.28, 1.49–12.33
India 4 [2009] Cauldbeck (64)	Cross-sectional study	53 patients	Self-reported missing of medications; 19% missed medications in the last week, 30% in the last month	41.5% university education; 47.7% total family income: 5,000-19,999 Rs/month	Education level: NS; Family income: NS
India 5 [2009]Naik (65)	Cross- sectional study; 2 hospitals, Mumbai	152 patients, on ART from 6 months to 5 years	Self-reported adherence assessment; 30% missing medication over a week	53% completed high school; 75% had ever missed medication because of the cost of treatment	Non-adherence: Cost of HAART
India 6 [2010]Batavia (66)	Cross- sectional study and medical chart review; Tertiary-care HIV clinic-based in Chennai	635 HIV patients	Self-reported 3 day-dose; Adherence rates of 95% or greater on 3-day recall were achieved by 84.6% of Tier 1 (n=156)	Secondary education: 33.3%; Monthly income: Median US$51.1	Education; Free medication
India 7 [2010] Lal (67)	Cross- sectional study	300 patients	Self-report; 75% adherence (not having missed even a single pill over the previous 4-day period)	53.7% employed; 43.7% <5 years of schooling	Non-adherence: Pay out-of-pocket for HAART: OR= 7.7, 3.9-15.1; Education: NS; Employment status: NS
India 8 [2010] Venkatesh (68)	Medical chart review data; Chennai	198 patients on HAART for at least 3 months	Self-report from the 30-day visual analogue scale in the past month. 31.8% reported 90% HAART adherence in the past month	Currently employed: Men: 94.9%; Women: 45.8%	Employment status: NS
Papua New Guinea [2010]Kelly (69)	Cross- sectional study, 6 provinces in PNG	374 HIV-positive people	Self-reported adherence in the past week; 62% complete adherence (no missed or late doses in the past week)	Elementary/primary education: 52%; Garden work employment: 42%	Education level: AOR=2.18, 1.05-4.54; Employment type: NS
Thailand [2010]Li (70)	Cross- sectional study	386 patients	Self-report; Among the 121 who reported failing to adhere to ART, 40.5% reported failing to adhere to ART in the past month	<High school education: 85.4%; Employed: 84.5%; Personal income: ≤35,000 Baht: 69.2%	Education: NS; Employment: NS

95% Confidence intervals; AOR=Adjusted odds ratio; NS=Not significant; OR=Odds ratio; RH=Risk Ratio

**Annexure F.** Impact of socioeconomic factors on HAART adherence among adults: study characteristics (Latin-America and Carribean 1)

Country of study, year of publication, first author (Reference number)	Study design, setting	Study population, sample-size, type of medication	Adherence: measurement, definition, and total adherence	Socioeconomic status	Impact of SES on adherence
Brazil 1 [2002] Pinheiro (71)	Cross- sectional study	195 patients aged 13 years or above	Self-reported in the previous 48 h; 56.9% reported ≥95% adherence on the previous two days	Years of schooling: Median 5 years; Monthly family income of <US$ 225: 73.8%	8 years of schooling vs 0–4: AOR=2.26, 1.02–5.02; Monthly income: NS
Brazil 2 [2004] Nemes (72)	Cross- sectional study; 60 health services sites	1972 outpatients on ART at least for 2 months	Self-reported adherence is the past 3 days; 75% adherent (≥95%)	Education (years): 8 or more: 45.6%; 0-2 years: 30%	Non-adherence; 0-2 years of schooling: AOR=1.48, 1.16-1.89
Brazil 3 [2007] de Carvalho (73)	Case-control study	105 patients; 35 non-adherent cases; 70 adherent controls	Self-reported assessment; 66.7% adherent	Incomplete primary education: 45.7%; Mean family income: 1587 Brazilian Real	Education: AOR=22.8, 1.9-270.9; Familial income: NS
Brazil 4 [2007] Seidl (74)	Cross- sectional study	101 HIV+ adults, ranging from 20 to 71 years of age (Mean=37.9 years)	Adherence was measured by self-reported number of ART pills/capsules missed during the previous week and previous month; 72.3% reported adherence of >95%	Incomplete primary education: 26.7%	Education: NS
Brazil 5 [2009] Blatt (75)	Cross- sectional study	67 patients	Self-reported dosage forgotten on the last day (70%); in three (76.1%) days; in seven (80.5%) days; and in fifteen (80.5%) days	Education (4-7 years): 46.3%	Educational level: NS
Brazil 5 [2009] Silva (76)	Cross- sectional study; outpatient clinics of 3 reference hospitals, Recife	412 patients; 67% on ART in previous 3 years	Self-reported assessment. 25.7% non-adherence (<90% of the total number of prescribed ART medication in the previous 5 days)	Less than 9 years of schooling: 51%; Family income <4 minimum wages: 62%	Higher income: AOR=2.33, 1.17–4.66; 8 years of schooling vs 11 years: NS
Brazil 6 [2010] Campos (77)	Longitudinal study; 2 public referral centres, Belo Horizonte	293 patients; Mostly two nucleoside reverse transcriptase inhibitors (NRTI) plus one non-nucleoside reverse transcriptase inhibitor (NNRTI)	Self-reported in the past 3 days; The overall cumulative incidence of non-adherence (<95%) was 37.2%,	Education <8 years: 49%; No income: 40.3%; Unemployed: 35.1%	Non-adherence; Low education: RH=1.71, 1.14-2.56; Unemployment: RH=1.90, 1.01-3.57; Monthly income: NS
Columbia [2009] Arrivillaga (78)	Cross- sectional study, 5 cities	269 women	Self-reported 21-item treatment adherence questionnaire; 43% of the women presented low (21-61 points on a scale from 21 to 84) adherence to treatment	Low social position (residence, SES, education, type of healthcare plan, occupation profile, income): 80%	Non-adherence; Member of subsidized national healthcare plan, or uninsured: OR=3.45, 1.96–6.18; Low social position: NS
Costa Rica [2004] Stout (79)	Cross- sectional study	88 patients	Self-reported 3-day adherence; 85% reported 100% adherence (not missing any) in the past 3 days	Post-secondary education: 54%; Work for pay: 32%	Education level: NS; Work for pay: NS
Cuba [2011] Aragonés (80)	Cross- sectional study; 25.1% in-patients, 74.9% in ambulatory care	1986 HIV-positive individuals	Self-reported number of doses taken in the past three days; 80.6% (≥95.0%) adherent	32.9% high school; 39.2% junior high school	Education: NS
Dominican Republic [2010] Harris (81)	Cross- sectional study	300 patients	Self-reported adherence; 24% suboptimal adherence in the past month	Less than high school education: 73%; Employed: 47%	Education: NS; Employment status: NS
Jamaica [2007] Williams (82)	Cross- sectional study	101 patients	Self-reported adherence; Mean adherence to tablets: 87.7%.	Employed: 50.5%; Secondary education: 60.2%	Employment status: NS; Level of education: NS

95% Confidence intervals; AOR=Adjusted odds ratio; NS=Not significant; OR=Odds ratio; RH=Risk ratio
